# State-of-the-Art Multimodality Imaging in Sudden Cardiac Arrest with Focus on Idiopathic Ventricular Fibrillation: A Review

**DOI:** 10.3390/jcm11164680

**Published:** 2022-08-10

**Authors:** Lisa M. Verheul, Sanne A. Groeneveld, Feddo P. Kirkels, Paul G. A. Volders, Arco J. Teske, Maarten J. Cramer, Marco Guglielmo, Rutger J. Hassink

**Affiliations:** 1 Department of Cardiology, Division Heart & Lungs, University Medical Center Utrecht, Heidelberglaan 100, 3584 CX Utrecht, The Netherlands; 2 Department of Cardiology, Maastricht University Medical Center, P. Debyelaan 25, 6229 HX Maastricht, The Netherlands

**Keywords:** sudden cardiac arrest, idiopathic ventricular fibrillation, multimodality imaging, electrocardiographic imaging

## Abstract

Idiopathic ventricular fibrillation is a rare cause of sudden cardiac arrest and a diagnosis by exclusion. Unraveling the mechanism of ventricular fibrillation is important for targeted management, and potentially for initiating family screening. Sudden cardiac arrest survivors undergo extensive clinical testing, with a growing role for multimodality imaging, before diagnosing “idiopathic” ventricular fibrillation. Multimodality imaging, considered as using multiple imaging modalities as diagnostics, is important for revealing structural myocardial abnormalities in patients with cardiac arrest. This review focuses on combining imaging modalities (echocardiography, cardiac magnetic resonance and computed tomography) and the electrocardiographic characterization of sudden cardiac arrest survivors and discusses the surplus value of multimodality imaging in the diagnostic routing of these patients. We focus on novel insights obtained through electrostructural and/or electromechanical imaging in apparently idiopathic ventricular fibrillation patients, with special attention to non-invasive electrocardiographic imaging.

## 1. Introduction

Sudden cardiac arrest (SCA) continues to be an international health problem and can be a fatal outcome of several cardiac diseases [1]. SCA is generally caused by ventricular arrhythmias, especially ventricular fibrillation (VF) [2]. Finding the underlying substrate causing VF might be challenging but is vital for targeted treatment and, potentially, for risk assessments of the family members of the patient. SCA survivors undergo extensive clinical testing, revealing coronary artery disease (CAD) as the leading cause in adults and leaving only a small subset of cardiac arrest survivors undiagnosed. When this work-up fails to identify a substrate for VF the diagnosis of idiopathic ventricular fibrillation (IVF) is made [3]. The prevalence of IVF varies between cohort studies (1.2% vs. 6.8% in two studies focusing on SCA patients with a shockable rhythm or cardiac cause) and patients tend to be younger compared to patients with an identifiable cause for SCA [4,5]. Advanced imaging modalities, drug provocation tests and genetic testing provide additional opportunities for the identification of an etiology, but a diagnosis remains elusive in an important subgroup of VF patients. We review the role of multimodality imaging in the diagnostic work-up for SCA and focus on the option to identify new subgroups and improve the detection of arrhythmogenic substrates.

## 2. Defining Idiopathic Ventricular Fibrillation

### 2.1. Background

**Figure 1:**
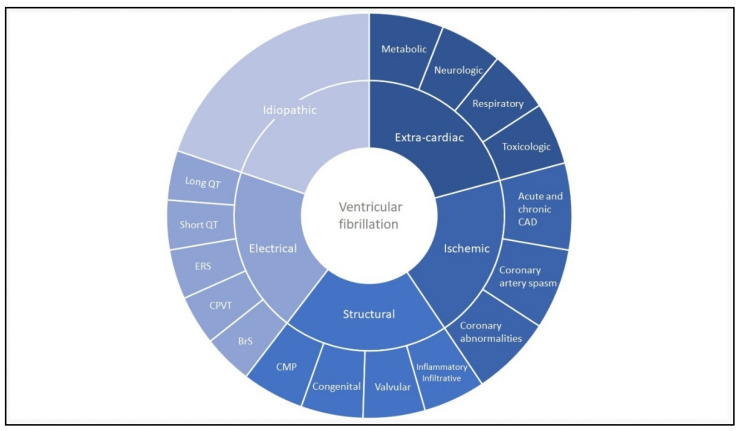
**Ventricular fibrillation: disease overview.** Most common causes of ventricular fibrillation stratified between extra-cardiac, ischemic, structural, electrical and (after exclusion) idiopathic ventricular fibrillation. The figure does not represent percentages. Cardiomyopathies most commonly include arrhythmogenic cardiomyopathy, dilated cardiomyopathy and hypertrophic cardiomyopathy. Inflammatory or infiltrative diseases include myocarditis, sarcoidosis and amyloidosis. Coronary abnormalities include coronary anomalies and myocardial bridging. BrS: Brugada syndrome; CAD: coronary artery disease; CMP: cardiomyopathy; CPVT: catecholaminergic polymorphic ventricular tachycardia; ERS: early repolarization syndrome; VF: ventricular fibrillation.

When we re-evaluate the first described cases of idiopathic ventricular fibrillation, possibly dating back to 1929, it is questionable if they would have received the same diagnosis today [6]. Many cases would now be considered primary arrhythmia syndromes or other, up to then unknown disorders. The growing expertise to identify underlying genetic disorders as a cause of VF increased the diagnosis of both primary arrhythmia syndromes and structural diseases in SCA survivors. The specific discovery of primary arrhythmia syndromes provided a diagnosis for patients with VF and a structurally normal heart; further expanding the previously considered differential diagnosis in IVF patients [7,8]. Besides primary arrhythmia syndromes, structural cardiac abnormalities enfold another part of the differential diagnosis in SCA. Structural cardiac abnormalities leading to VF include cardiomyopathies, myocarditis, sarcoidosis, amyloidosis, valvular and congenital heart diseases [1,3]. Diagnostic criteria to distinguish and identify different cardiomyopathies are continuously improving with the ongoing evolution of the non-invasive cardiac imaging [9]. These imaging modalities facilitate the detection of structural abnormalities in SCA survivors. Figure 1 provides an overview of the spectrum of VF substrates. The broad differential diagnosis that needs exclusion before diagnosing a patient with IVF requires a thorough clinical investigation, including the use of multimodality imaging.

### 2.2. Clinical Evaluation

**Figure 2:**
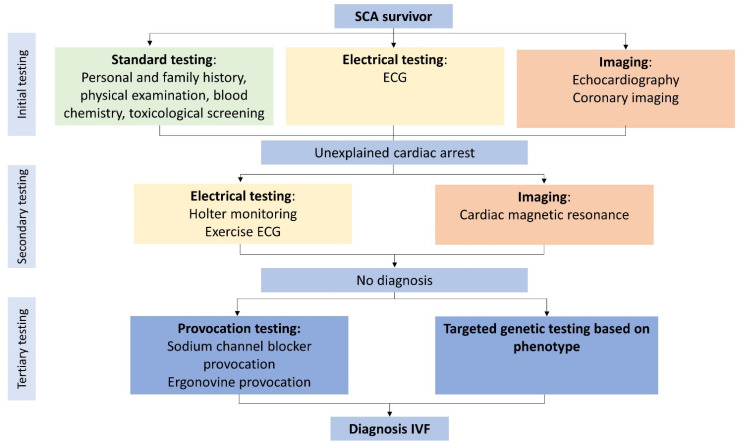
**Minimal diagnostic tests required before diagnosing idiopathic ventricular fibrillation.** Flowchart with all diagnostic tests for excluding potential causes of ventricular fibrillation. Coronary angiography can be replaced with non-invasive coronary imaging in young, low-risk patients. If a diagnosis is lacking after initial testing, it is considered an unexplained cardiac arrest. ECG: electrocardiogram; IVF: idiopathic ventricular fibrillation; SCA: sudden cardiac arrest. Adapted with permission from Visser et al. [3] and Wolters Kluwer Health, Inc., 2016. The Creative Commons license does not apply to this content. Use of the material in any format is prohibited without written permission from the publisher, Wolters Kluwer Health, Inc.

The 2013 Heart Rhythm Society (HRS)/European Heart Rhythm Association/Asia Pacific Heart Rhythm Society (APHRS) expert consensus statement defines IVF as “resuscitated cardiac arrest, preferably with documentation of VF, for which known cardiac, respiratory, metabolic, and toxicological etiologies have been excluded through clinical evaluation”. Or as stated in 1997 “the terminology that best acknowledges our current inability to identify a causal relationship between the clinical circumstance and the arrhythmia” [10,11]. To gain more certainty about the clinical circumstances affecting the arrhythmia, Visser et al. proposed a flowchart including all diagnostic tests needed before diagnosing IVF (Figure 2) [3]. This flowchart standardized the diagnostic approach for IVF patients. Just recently, an expert recommendation regarding the value of different diagnostic tests for SCA survivors was published in the 2020 APHRS/HRS expert consensus statement [12]. Based on the flowchart from Visser et al., the initial clinical assessment of SCA survivors should contain blood chemistry (including toxicological screening), chest X-ray, electrocardiography (ECG), echocardiography, coronary angiography (CAG) or non-invasive coronary imaging with computed tomography (CT) in young patients with a low risk for CAD. When the initial tests do not lead to a diagnosis, secondary diagnostics include exercise testing, Holter or telemetry monitoring and cardiac magnetic resonance imaging (CMR). Finally, provocation test for Brugada syndrome and coronary artery spasm, and targeted genetic testing based on phenotype should be performed before diagnosing a patient with IVF. Since the publication of the flowchart of Visser et al., several studies have suggested an equivalent diagnostic approach [13,14,15,16]. All diagnostic approaches incorporate multimodality imaging. SCA survivors undergo an initial diagnostic assessment to rule out evident causes for the arrest. Cardiac arrest is considered unexplained (UCA) when no abnormalities are revealed during this initial testing. Previous studies in UCA survivors (Table S1) show a diagnostic yield for CMR of 10–35% in patients left unexplained after initial testing [4,17,18]. The diagnostic yield of CMR determined in SCA survivors before initial testing is higher [19]. The initial work-up influences the diagnostic yield of the additional tests, resulting in different yields between SCA survivors (with limited diagnostic testing) compared to UCA survivors. When reviewing available cohort studies focusing on either UCA or IVF patients, it stands out that the performed diagnostic work-up is often not complete (Tables S2 and S3) [4,5,17,18,20,21,22,23,24,25,26,27,28]. An incomplete diagnostic work-up leads to an inconsistent use (and possible overuse) of the terminology IVF in current literature [15]. To prevent an erroneous IVF diagnosis in patients with an underlying disease it is of utmost importance to fulfill a complete workup.

## 3. The Use of Multiple Imaging Modalities

Non-invasive imaging is a well-implemented part of the evaluation of SCA survivors and enables the detection of several structural diagnoses. The following sections of this review provide an overview of the yield of different imaging modalities during the evaluation of SCA survivors.

### 3.1. Echocardiography

The diagnostic use of echocardiography already starts during the acute phase of cardiac arrest, as it is able to identify signs of several acute and potentially reversible causes. Echocardiography can detect cardiac tamponade, CAD (wall motion abnormalities) and secondary signs of acute pulmonary embolism or hypovolemia [29]. After the acute phase, echocardiography is incorporated in the standard initial evaluation following a cardiac arrest, along with an ECG and CAG or coronary CT. Conventional 2D-echocardiography can be seen as a proper screening tool and a sophisticated first evaluation of SCA survivors [12]. It is inexpensive, easily available, and enables the diagnosis of valvular and congenital heart diseases associated with SCA [30]. In case of an inconclusive ECG after resuscitation, echocardiography can reveal regional wall motion abnormalities which raise suspicion for CAD [31]. Regional wall motion abnormalities are more frequently seen in SCA survivors with CAD than without (64% vs. 18%), as shown by Lee et al. [32]. It can be difficult to differentiate between acute or chronic CAD and other cardiac diseases that present with wall motion abnormalities if only echocardiography is used. In inflammatory diseases (myocarditis, sarcoidosis), echocardiography can show additional findings typical for each disease, but with limited sensitivity [33,34,35]. A systematic review focusing on the diagnostic yield of non-invasive imaging modalities in SCA survivors showed that overall, echocardiography determined regional wall motion abnormalities in 28.6% of 241 patients [36]. Waldman et al. showed that with only the initial investigation using ECG, coronary imaging and echocardiography, a diagnosis can be established in approximately 88% of SCA survivors [4]. Echocardiography is an important and well-established part of the diagnostic pathway for cardiomyopathies. Individual patients with cardiomyopathy can show overlapping phenotypes, complicating the diagnosis with only echocardiography. Especially in arrhythmogenic cardiomyopathy (ACM), echocardiographic diagnosis is challenging and requires expertise [37,38,39]. Importantly, the cardiac arrest itself can cause myocardial stunning, which is visible as global dysfunction that resolves within 48–72 h. This indicates the importance of repeating the echocardiography after this time interval [40,41].

#### Focus on Idiopathic Ventricular Fibrillation

**Figure 3:**
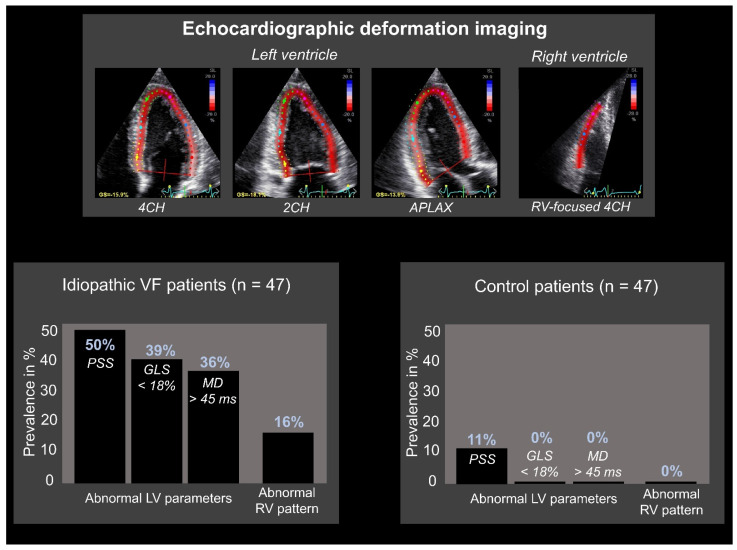
**Echocardiographic deformation imaging in patients with idiopathic ventricular fibrillation.** Abnormal results were primarily found in idiopathic ventricular fibrillation (IVF) patients compared to healthy controls. Global deformation imaging includes left ventricular (LV) global longitudinal strain (GLS): defined as “the average global peak strain from three apical views” and LV mechanical dispersion (MD): defined as “the standard deviation of time to peak longitudinal strain from the 18 LV segmental deformation curves”. LV GLS < 18% was considered abnormal, LV MD > 45 ms was considered abnormal. Regional deformation imaging includes postsystolic shortening (PSS): defined as “longitudinal myocardial shortening after aortic valve closure”. PSS ≥ 10% was considered abnormal. 2CH: 2-chamber; 4CH: 4-chamber; APLAX: apical long axis; RV: right ventricular. Adapted with permission from Groeneveld et al. [45].

**Figure 4:**
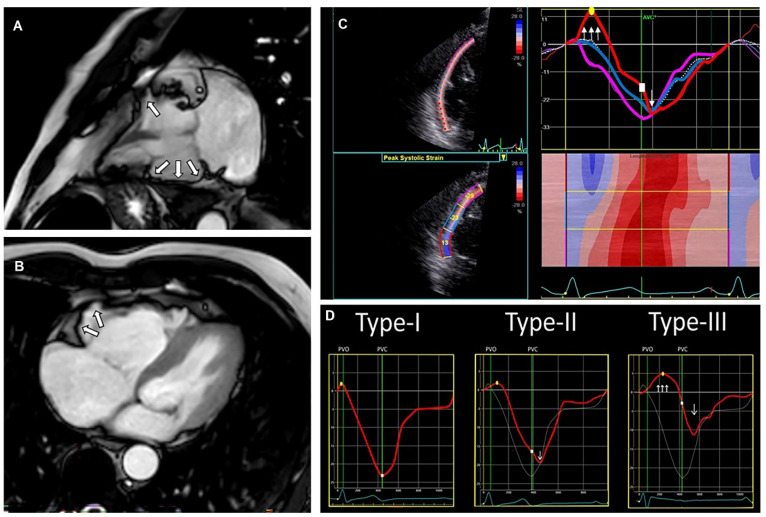
**Cardiac magnetic resonance (CMR) images from a patient with arrhythmogenic cardiomyopathy (ACM), including a typical echocardiographic deformation imaging pattern.** CMR images (**A**,**B**) with right ventricular (RV) aneurysm (white arrows). Echocardiographic deformation imaging (**C**) with RV focused and peak systolic strain measurements and deformation pattern. Distinct types of RV deformation patterns (**D**) as determined by Mast et al. [47]; type-I: normal pattern; type-II: “delated onset of shortening and decreased systolic peak strain”; type-III: “systolic stretching and passive recoil or shortening during early diastole”. The patients’ strain pattern shows a type-III strain pattern, fitting structural ACM.

Concealed cardiomyopathies contribute to a part of the underlying etiology of IVF patients, as shown both on genetic and clinical basis [42,43]. One method to reveal a concealed stage of disease is echocardiographic deformation imaging, which provides the quantification of myocardial deformation. It has emerged in clinical use during the last decade since it has shown the ability to detect early stages of cardiac diseases, like genetic cardiomyopathies or possibly inflammatory diseases [33,44]. Investigating patients diagnosed with IVF (after adequate clinical assessment as described above) with additional echocardiographic deformation imaging could unravel supplementary findings that lead to an alternate diagnosis. Groeneveld et al. compared 47 IVF patients with 47 healthy controls (age and sex-matched) and analyzed echocardiograms with deformation imaging by 2-dimensional speckle tracking (the most commonly used tool) [45,46]. The authors showed both global and regional echocardiographic deformation abnormalities primarily in IVF patients (Figure 3). Global deformation imaging revealed systolic dysfunction, possibly due to diffuse myocardial damage caused by global hypoxemia during the performed resuscitation. However, it could also reflect early-stage cardiac disease. In addition, the difference in left ventricular mechanical dispersion, a measure of the heterogeneity of myocardial contraction, may represent subtle fibrosis. Again, this could have been induced by the previous cardiac arrest, but could also be a sign of unrecognized cardiac disease [45]. Additional studies are necessary to investigate these findings in larger patient cohorts and discover the exact underlying etiology. Discriminating between systolic dysfunction as the cause or the consequence of VF is complicated. Besides global deformation abnormalities, regional deformation abnormalities were also found. It should be further investigated if these abnormalities are caused by structural or localized electrical abnormalities [45]. Last, when focusing on right ventricular deformation imaging with three distinct deformation patterns previously characterized by Mast et al. (Figure 4), abnormal patterns were again more abundant in IVF patients [47]. Other emerging technologies in echocardiographic imaging are, to the best of our knowledge, not investigated in IVF cohorts, limiting the focus of this review to deformation imaging.

### 3.2. Cardiac Magnetic Resonance

After echocardiography, CMR is indicated in all UCA patients [12]. CMR serves as the reference standard for the assessment of chamber volumes and mass, wall motion abnormalities and contractile function. It particularly increases the accuracy of right ventricular volumetric measurements compared to the echocardiography [30,39]. Furthermore, CMR enables cardiac tissue characterization with late gadolinium enhancement (LGE), T1- and T2-mapping, identifying either fibrosis, fat, or edema [48]. Specific patterns of LGE can help determine the etiology, differentiating between ischemic, genetic or inflammatory diseases [49]. The diagnostic value of CMR in SCA survivors is well studied and repeatedly proven to be beneficial [19,50,51,52,53,54,55,56,57,58,59]. The ability to identify a substrate in SCA survivors with CMR was first demonstrated by White et al., providing a diagnostic yield of 63% [19]. An overview of cohort studies focusing on SCA survivors and the ability to identify a pathological substrate with CMR can be found in Table S4. Compared to echocardiography, CMR more frequently identified a pathological substrate (69% vs. 54%), primarily caused by LGE assessment [54]. Neilan et al. showed that LGE is present in 71% of UCA survivors. LGE (both the presence and extent) proved to be a predictor for future adverse events (adjusted hazard ratio presence: 6.7 (95% CI: 2.38–18.85), extent: 1.15 (95% CI 1.11–1.19) [50]. Some LGE patterns are, while not exclusive, suggestive for specific diagnoses; with midwall LGE fitting dilated cardiomyopathy (DCM) and hypertrophic cardiomyopathy (HCM), subepicardial LGE inflammatory diseases and subendocardial (with coronary distributions) LGE ischemic cardiac disease [60,61]. In contrast, findings of myocardial edema might suggest a higher survival free of arrhythmic events during follow-up, indicating the association of myocardial edema with an acute and transient arrhythmogenic substrate. However, these results need further investigation [51]. Correlating edema to specific LGE patterns will enable the differentiation between acute ischemic disease (subendocardial pattern) and acute inflammatory disease (subepicardial pattern) and might indicate the need for revascularization or additional testing with ergonovine provocation for coronary spasm [19]. Two recent studies are only showing subtle abnormalities with CMR imaging. These results were based on specific subgroups of SCA survivors, and the authors did present minor abnormalities discovered with the use of CMR that could be fitting with early remodeling (Table S4) [55,56]. Frequent diagnoses made with CMR were an ischemic cardiac disease, acute myocarditis, ACM, DCM, and HCM. Combining CMR with new imaging techniques such as echocardiographic deformation imaging is useful for diagnosing structural heart diseases. Figure 4 provides an example of the use of CMR and echocardiographic deformation imaging in the diagnosis of ACM. Besides ischemic cardiac disease and cardiomyopathies, valvular abnormalities are a potential cause of VF. Mitral valve prolapse (MVP) is a valvular abnormality that has been associated with sudden cardiac death [62]. While echocardiography is well suited for diagnosing MVP, CMR can advance the identification of an arrhythmogenic substrate [62,63,64]. MVP is defined by superior displacement of one or both leaflets of the mitral valve into the left atrium [65]. It is suggested that prolapsing mitral valve leaflets induce myocardial stretch of the papillary muscles and inferolateral left ventricular wall, resulting in the formation of fibrosis. This fibrosis might be the arrhythmogenic substrate in MVP and is identifiable with LGE [62]. Besides LGE, focal and diffuse fibrosis in patients with MVP could also be identifiable with T1-mapping and myocardial extracellular volume quantification, as shown by Guglielmo et al. [63] and Pavon et al. [64]. Applying these two techniques reduces the use of contrast infusion in CMR. Fibrosis of the papillary muscles might be influenced by the insertion of the papillary muscles. Moura-Ferreira et al. showed that papillary fibrosis was more prevalent in MVP patients with an apical insertion. In addition, the authors showed more premature ventricular contractions and non-sustained ventricular tachycardias in MVP patients with an apical insertion, suggesting a possible influence on the arrhythmogenic substrate [66]. However, more research is necessary. In conclusion, CMR is of utter importance to detect a possible arrhythmogenic substrate in UCA survivors and provides valuable information in addition to echocardiography.

#### Focus on Idiopathic Ventricular Fibrillation

**Figure 5:**
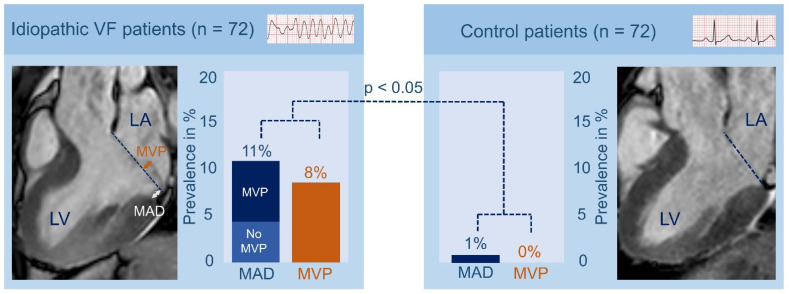
**Prevalence of mitral valve prolapse and mitral annulus disjunction in idiopathic ventricular fibrillation patients.** Mitral valve prolapse (MVP) and mitral annulus disjunction (MAD) were significantly more prevalent in idiopathic ventricular fibrillation patients compared with control patients. MVP is defined as “the displacement of >2 mm of one or both leaflets beyond the annular hinge points at end systole”; MAD is defined as “longitudinal displacement of >1 mm”. LA: left atrium; LV: left ventricle; VF: ventricular fibrillation. Adapted with permission from Groeneveld et al. [67].

Re-evaluating the diagnosis of IVF in patients based on the expanded medical knowledge and advanced diagnostic techniques will eventually lead to the discovery of unidentified pro-arrhythmic factors. Hence, Groeneveld et al. re-evaluated CMR images to study the prevalence and morphology of mitral annulus disjunction (MAD) and MVP in a multicenter IVF cohort. Mitral valve abnormalities have been previously associated with UCA [67,68]. MAD is a structural abnormality in which the hinge point of the mitral valve is abnormally placed in the atrium [69]. Previous studies have reported a high prevalence of MVP in patients with UCA, but until recently no specific attention was given to the presence of MAD in IVF patients [68]. The study of Groeneveld et al. revealed that inferolateral MAD and MVP were significantly more prevalent in IVF patients compared to healthy controls (Figure 5). The authors recommended that MAD and MVP should deserve specific attention when evaluating UCA patients and showed the value of re-evaluating IVF patients based on the expanded medical knowledge during follow-up [67].

### 3.3. Computed and Positron Emission Tomography

#### 3.3.1. Cardiac Computed Tomography

**Figure 6:**
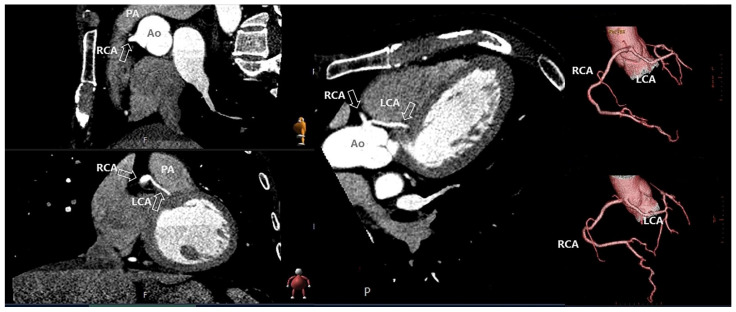
**Coronary anomaly visible and diagnosed with computed tomography.** The figure shows the anomalous origin of the left coronary artery (LCA) from the right sinus of Valsalva with the LCA between the aorta and the pulmonary artery. Ao: aorta; PA; pulmonary artery; RCA; right coronary artery.

Cardiac computed tomography (CT) can accurately quantify ventricular volume, mass and function, showing a good correlation with CMR. It is, however, less frequently performed in SCA survivors and not recommended in consensus statements for the cardiac evaluation of SCA survivors or for diagnosing cardiomyopathies due to radiation exposure [12,30,37,38,39]. Cardiac CT can be used when echocardiographic imaging is suboptimal and CMR imaging is contra-indicated. Tissue characterization (fibrosis) with CT is possible with late iodine enhancement and the assessment of extracellular volume. These techniques show similar results compared to CMR localized fibrosis using either LGE or extracellular volume [70,71]. A low signal-to-noise ratio and high dosages of contrast are limiting the evaluation of fibrosis with cardiac CT. Still, the ability of tissue characterization is an emerging role of CT and of particular value for SCA survivors. When a diagnosis is not fulfilled before implanting an implantable cardiac device, it creates a contra-indication for CMR when the device is not compatible with CMR. In addition, compatible devices can induce artifacts, hampering the identification of a diagnosis or substrate for VF [72]. Furthermore, during the preparation of VT-ablation, late enhancement in CT might be of value [73]. With CAD as the leading cause of SCA, it is important to visualize the coronary arteries. Coronary CT serves as a good alternative for CAG in low-risk, young (<45 years) patients to identify CAD [74,75]. Moreover, it can be used to determine anomalous coronary circulation. CT is superior to invasive CAG for determining high-risk anatomic features of coronary anomalies, associated with SCA [76]. Figure 6 shows an example of a coronary anomaly diagnosed with CT. Indeed, not solely cardiac causes can induce an arrest and extra-cardiac causes are necessary to rule out. In comatose patients, an early head CT or pulmonary artery CT should be considered (before or after CAG, depending on the likelihood of a cardiac cause) before admitting a patient to the intensive care unit [77]. In the systematic review of Petek et al., head CT, chest CT or abdominal CT revealed potential causes of SCA in 8–54% of the performed CT-scans. Common findings included brain hemorrhage, acute stroke and pulmonary embolism. The exact role of CT for excluding extra-cardiac causes in SCA needs further investigation [36]. Besides the role of CT in coronary imaging, cardiac CT is not incorporated in the standard evaluation of SCA survivors [12]. However, the combination of CT with positron emission tomography (PET) or electrocardiographic imaging (ECGI) could provide additional findings in SCA survivors.

#### 3.3.2. Positron Emission Tomography

**Figure 7:**
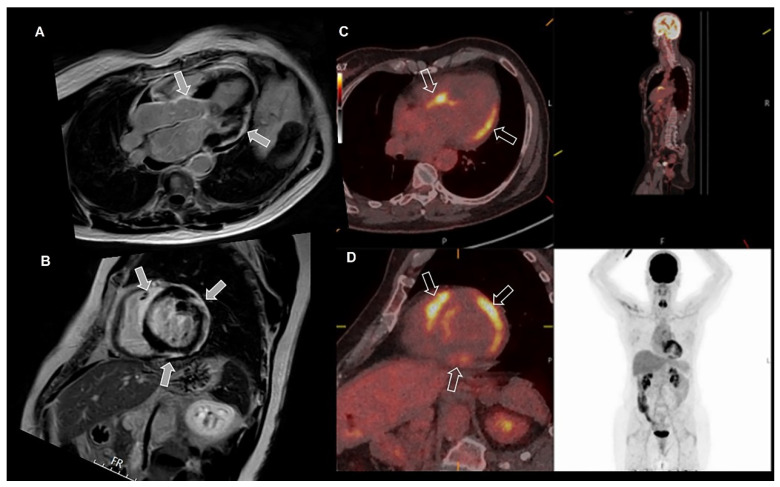
**Cardiac sarcoidosis diagnosed with cardiac magnetic resonance (CMR), positron emission tomography (PET) and computed tomography (CT).** The figure shows the presence of subepicardial late gadolinium enhancement inferior and lateral, and midmyocardial in the basal interventricular septum (arrows), suspicious for sarcoidosis on CMR (**A**,**B**). PET-CT images (**C**,**D**) show corresponding increased uptake (arrows) of fluorodeoxyglucose, indicating inflammatory lesions associated with sarcoid activity in the corresponding areas, enabling the diagnosis of sarcoidosis. Note the absence of tracer uptake outside the heart indicating isolated cardiac sarcoidosis.

When CMR reveals abnormalities consistent with sarcoidosis (subepicardial edema, LGE); PET-CT imaging is indicated and is a competent imaging modality to help diagnose sarcoidosis. PET-CT can visualize a specific pattern for cardiac sarcoidosis with patchy uptake [12,33,34]. Figure 7 shows an example of cardiac sarcoidosis, suspected after CMR, and confirmed with PET-CT. Whole-body PET-CT also entails the possibility to detect extra-cardiac inflammation. However, the gold standard for diagnosing inflammatory or infiltrative diseases such as myocarditis, sarcoidosis, or amyloidosis remains a biopsy with histopathological evidence of disease [34,78,79].

#### 3.3.3. Focus on Idiopathic Ventricular Fibrillation

Compared to echocardiography and CMR, cardiac CT is less frequently performed in diagnosed IVF patients [4,5,21]. With this infrequent use of cardiac CT in IVF cohorts, the diagnostic value remains unknown. Despite that, cardiac CT fulfills a sufficient role in the reconstruction of cardiac electrical activity in ECGI. ECGI is currently emerging and may be proven useful for identifying pro-arrhythmic substrates and risk prediction of SCA [80].

### 3.4. Reconstruction of Cardiac Electrical Activity

The 12-lead ECG has sufficiently improved our understanding of the electrical activity in the heart by measuring an attenuated, dispersed reflection of the electrical signals from the heart on the body surface. The possible role of an invasive electrophysiological study in the diagnostic work-up of SCA patients, when other imaging modalities reveal no abnormalities, is described in a review by Haïssaguerre et al. [14]. They discuss their findings of combining high-density invasive mapping and non-invasive mapping in IVF patients. This study showed that localized myocardial alterations can be revealed with invasive high-density mapping of IVF patients when CMR imaging of the same areas did not reveal any abnormalities [14]. The results from this study show the possible additional value of adding invasive high-density mapping and/or ECGI to the diagnostic work-up of IVF patients. Current guidelines state that an invasive electrophysiological study to evaluate a substrate may be considered in SCA survivors when no cause is identified [12]. This section will further focus on the non-invasive reconstruction of cardiac electrical activity.

#### 3.4.1. Non-Invasive Reconstruction: ECG-Imaging

**Figure 8:**
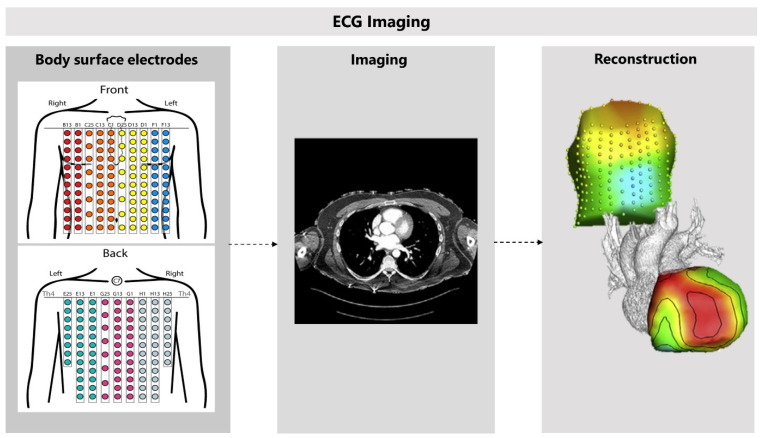
**The use of cardiac computed tomography in electrocardiographic imaging.** Electrocardiographic imaging (ECGI) starts with recording body-surface ECGs, using 256 electrodes. Cardiac computed tomography is used to create a patient-specific heart-torso geometry. Epicardial potentials can be mathematically reconstructed when combining these results. Adapted with permission from Cluitmans et al. [84] and permission from Elsevier, 2018.

ECGI facilitates the non-invasive measurement of the electrical activity at the level of the heart muscle by mathematically reconstructing these signals from an array of body-surface ECGs and a patient-specific heart-torso geometry (Figure 8) [81]. The patient-specific heart-torso geometry is created with anatomical imaging, using either cardiac CT or CMR imaging. ECGI can reconstruct electrograms, potential maps, and activation or recovery isochrones. However, validating ECGI continues to be a challenge [82]. Several limitations of ECGI are complicating the validation and the incorporation into the clinical practice [83]. For example, implementation choices and the type of application for which ECGI is used can largely influence ECGI and complicate the validation [82]. Moreover, performing ECGI can be difficult and requires adequate training of personnel, for both the measurements and interpretations of ECGI [83]. Keeping these shortcomings in mind, we will address the possible applications for ECGI, and discuss the first results of ECGI in IVF patients.

#### 3.4.2. Clinical Validation and Application

ECGI investigations for validation included computer models, torso-tank experiments and eventually in vivo validation [84,85]. Possible applications for ECGI entail atrial fibrillation characterization, identifying accessory pathways or substrates for VT, atrial tachycardias or myocardial infarction, optimizing cardiac resynchronization therapy and identifying repolarization abnormalities. It may also serve as a tool for risk stratification in SCA when able to discover repolarization and conduction abnormalities and identify scar tissue [82]. However, as mentioned, ECGI still needs further research and improvement before it can be implemented in the guidelines.

#### 3.4.3. Finding Pro-Arrhythmic Factors in Idiopathic Ventricular Fibrillation Patients

Conduction and repolarization abnormalities, and abnormal focal excitation, can serve as a substrate for arrhythmias without proven structural abnormalities [14]. A basis for VF could be created by regional differences in repolarization time, promoting re-entry of a premature ventricular beat arising in a region with an early repolarization time to a region with a late repolarization time. This dispersion of repolarization has long been associated with ventricular arrhythmias [86,87]. Subtle repolarization abnormalities are difficult to detect on a 12-lead ECG and might go unnoticed. Recent improvements in ECGI provide the possibility to non-invasively measure regional repolarization [88]. Cluitmans et al. investigated the hypothesis of a repolarization substrate promoting re-entry and resulting in VF in IVF patients [80]. The authors included IVF patients, patients with frequent monomorphic premature ventricular contractions (PVC) requiring catheter ablation and healthy control subjects. ECGI measurements were conducted in all patients. The study revealed differences in local repolarization time in all groups but with a higher prevalence in IVF patients. Secondly, IVF patients had large regions with early repolarization. Last, PVCs in the PVC patient group mainly arose from regions with late repolarization, whereas in IVF patients PVCs arose from regions with early repolarization. Ex-vivo testing showed that arrhythmias were only inducible with stimulation in early repolarization time regions, but not in late repolarization regions. Interestingly, the authors showed that repolarization intervals on a regular 12-lead ECG did not differ between the investigated groups, suggesting that these abnormalities cannot be perceived with standard diagnostic testing. However, given the small sample size, the hypothesis needs further proof in larger cohorts with IVF patients and more research is necessary for complete understanding all the factors contributing to a repolarization substrate. Increasing the ability to non-invasively detect repolarization abnormalities contributes to understanding the pathophysiology of IVF and could be promising for early detection of patients at risk for VF. Furthermore, ECGI serves the potential to improve the genotype-phenotype association in IVF patients and previously found genetic mutations (e.g., DPP6-haplotype and SCN5A in the Netherlands), in which the pathophysiology leading to VF is still not fully understood [89,90].

## 4. Multimodality Imaging Approach and Future Perspectives

**Figure 9:**
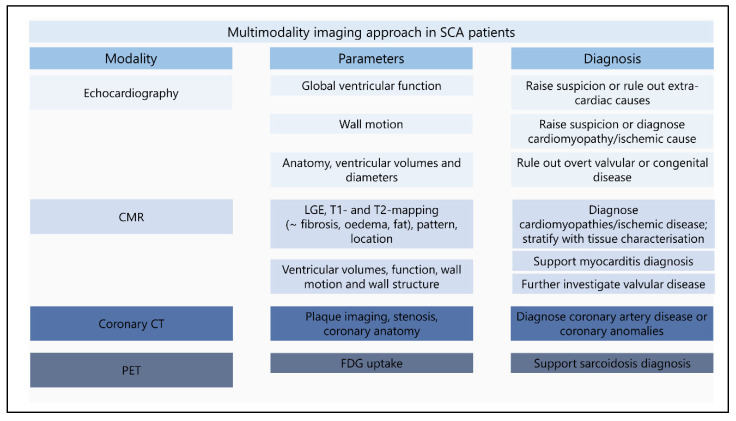
**Multimodality imaging approach in sudden cardiac arrest survivors.** The figure provides an overview of the minimum performed imaging modalities in sudden cardiac arrest survivors. Echocardiographic imaging is the first in line imaging modality, revealing a first clue for a structural abnormality or providing the diagnosis. CMR further determinates the abnormalities found during echocardiography and reveals additional findings with tissue characterization. Coronary CT and PET imaging can be used in specific patients for the diagnosis of coronary artery disease or sarcoidosis. New diagnostic techniques (deformation imaging and ECGI) should be implemented after further investigation. CMR: cardiac resonance imaging; CT: computed tomography; ECGI: electrocardiographic imaging; FDG: fluorodeoxyglucose; LGE: late gadolinium enhancement; SCA: sudden cardiac arrest; PET: positron emission tomography.

This review describes different imaging modalities proven or potentially useful for the identification of a substrate for VF. Figure 9 provides a multimodality imaging approach for SCA survivors with parameters and diagnoses stratified by imaging modality. It is important to emphasize that a diagnosis is not only based on imaging features and always needs to be combined with other diagnostic results. The start of identifying structural abnormalities in SCA survivors is relatively simple, abundant abnormalities are easily detectable and lead to known diagnoses. The challenge begins when only subtle abnormalities are found. The inclusion of new findings with advanced imaging techniques will result in additional abnormalities for which the etiology still needs to be elucidated. Combining different imaging modalities contributes to accomplishing the diagnosis. Further research is required to investigate the value of subtle and new findings in IVF patients. Large prospective studies are currently lacking. With the ECGI developments in combination with imaging modalities the possibility to identify patients at risk for SCA will hopefully become available.

## 5. Conclusions

Revealing the underlying etiology in SCA remains a challenge. The importance of a complete workup is vital for discovering a disease and improving management. Echocardiography and CMR should be performed on all UCA survivors. Coronary, cardiac, or PET-CT are indicated in specific cases. Promising new techniques are deformation imaging and ECGI, which might be implemented in the standard diagnostic assessment of IVF patients after further investigation.

## Supplementary Materials

The following supporting information can be downloaded at: https://www.mdpi.com/article/10.3390/jcm11164680/s1, Table S1: Additional diagnostic imaging yield in patients with unexplained cardiac arrest.; Table S2: Overview of cohort studies with unexplained cardiac arrest or idiopathic ventricular fibrillation patients; Table S3: Overview of the diagnostic work-up performed in unexplained cardiac arrest of idiopathic ventricular fibrillation cohorts; Table S4: Summary of studies focusing on CMR in patients with ventricular arrhythmias.
