# Supplementary material for: State-of-the-Art Multimodality Imaging in Sudden Cardiac Arrest with Focus on Idiopathic Ventricular Fibrillation: A Review
Source: J Clin Med. 2022 Aug 10;11(16):4680. doi: 10.3390/jcm11164680 (PMC9410297; doi:10.3390/jcm11164680)
Supplement: Supplementary file 1 [file jcm-11-04680-s001.zip › jcm-1843235-supplementary.pdf]

Supplementary tables

Table S1. Additional diagnostic imaging yield in patients with unexplained cardiac arrest.

First Author, Year, Journal	Setting	Study population	Clinical assessment	N	Baseline characteristics	Diagnostic imaging yield		
Herman, 2016, *Circulation A&E*[1]	Multicenter	Survivors of an unexplained CA with documented cardiac collapse or VT/VF requiring defibrillation. Patients with known causes* for CA are excluded.	All patients underwent standard care*, enrolled patients received additional testing if applicable (stress-ECG, saECG, CMR with gadolinium and provocation testing)	200	Age; 41.5±14.7 years, 41% female	CMR Performed 154/200 (77%)<br><br>Diagnostic yield 15/154 (9.7%)		Overall diagnostic yield 68/200 (34%) at baseline<br><br>81/200 (40.5%) during FU
Merghani, 2021, *J Am Heart Assoc*[2]	Single center	SCA survivors with documented VT or VF	Comprehensive cardiac testing#, with additional tests (CMR, Ajmaline testing, saECG, exercise-ECG, genetic testing) when inconclusive	327 SCA survivors<br>31 unexplained after initial systematic testing	Age; 61.9±16.2 years, female 20.2%	CMR Performed 24/31 (77%)<br>Diagnostic yield 3/24 (12.5%)	Echo performed 31/31(100%)<br>Diagnostic yield 1/31 (3.2%)	Overall diagnostic yield 15/31 (48%) during FU
Waldman, 2018, *European Heart Journal*[3]	Multicenter	SCA survivors	Cardiac testing at the discretion of the medical team after initial investigation†	717 SCA survivors<br>88 unexplained after initial testing	NR for all patients	CMR Performed 72/88 (82%)<br>Diagnostic yield 25/72 (34.7%)		Overall diagnostic yield baseline and FU 39/88 (44%)

* Causes: Patients were excluded in case of coronary artery disease (stenosis >50% or anomalous coronary arteries), hyperkalemia, drug overdose, hypertrophic cardiomyopathy, commotio cordis, Brugada syndrome or idiopathic ventricular tachycardia. Standard care included continuous ECG telemetry for 72 hours, echocardiography and coronary angiography or cardiac CT or MRI.

Comprehensive cardiac testing: history, physical examination, ECG, echocardiogram, and coronary angiography

† Initial investigation: ECG, echocardiogram, and coronary angiography.

Abbreviations: ECG: electrocardiography, CMR: cardiac magnetic resonance, FU: follow up, saECG: signal-averaged electrocardiography, NR: not reported, SCA: sudden cardiac arrest, VT: ventricular tachycardia, VF: ventricular fibrillation

Table S2. Overview of cohort studies with unexplained cardiac arrest of idiopathic ventricular fibrillation patients. *Studies focusing on genetic testing are not included.*

First Author, Year, Journal, Country	Setting	Patient cohort	N
Blom, 2019, *Europace*[4]	Multicenter<br><br>The Netherlands	Cardiac arrest patients with shockable rhythm and diagnosis of IVF	217
Conte, 2017, *Europace*[5]	Single Center<br><br>Switzerland	OHCA patients due to VT or VF (documented), considered as IVF after exclusion of cardiac, respiratory, metabolic or toxicological etiology and maintaining a normal ECG during FU	3407 OHCA patients, 2192 cardiac origin OHCA, 8 true IVF patients
Conte, 2019, *Europace* [6]	Multicenter study (web-based) in several countries:<br>Belgium, France, Germany, Greece, Israel, Italy, Mexico, Norway, Spain, Switzerland, United Kingdom	OHCA patients with IVF; normal ECG and echocardiography at baseline and follow up, exclusion of cardiac, respiratory, metabolic and toxicological etiology	245
Chaudhry, 2019, *Journal of Electrocardiology* [7]	Multicenter<br><br>Sweden	Cardiac arrest survivors with IVF	50
Cunningham, 2020, *Heart Rhythm.* [8]	Multicenter<br><br>Canada	UCA in children and adolescents (1-19 years), excluding known causes for cardiac arrest	46
Frontera, 2019, *JAHA* [9]	Multicenter<br><br>45 tertiary cardiac care centers worldwide	SCA with documented VF, absence of an underlying cause following comprehensive analysis, age ≤ 16 years	54
Herman, 2016, *Circulation A&E.* [1]	Multicenter<br><br>Canada	Survivors of an UCA with documented cardiac collapse or VT/VF requiring defibrillation. Patients with known causes are excluded	200
Jiménez-Jaimez, 2015, *American Journal of Cardiology* [10]	Multicenter<br><br>Spain	UCA patients (no diagnostic findings on ECG, echocardiography or CAG) with performed AED shock. Excluded known causes for arrest	35
Merghani, 2021, *J Am Heart Assoc* [2]	Single center<br><br>United Kingdom	SCA survivors with documented VT or VF	327 SCA survivors, 31 UCA after initial testing (ECG, echocardiography, CAG)
Peres, 2020, *Archives of Cardiovascular Diseases Supplements,* abstract[11]	Single center<br><br>France	VF survivors without reversible cause	66, 40 UCA after initial testing (ECG, echocardiography, CAG)

Stampe, 2020, *JCE* [12]	Single center<br><br>Denmark	SCA survivors (secondary ICD indication) and IVF diagnosis	1319 ICD indications, 81 IVF patients
Stepien-Wojno 2018, *Polish Archives of Internal Medicine*[13]	Single center<br><br>Poland	UCA survivors and family members, unexplained after exclusion of known causes	44
Waldman, 2018, *European Heart Journal*[3]	Multicenter<br><br>France	SCA survivors	717 cardiac SCA, 88 UCA after initial testing (ECG, echocardiography, CAG), 49 IVF patients

Abbreviations: AED; automated external defibrillator, CAG; coronary angiogram, ICD; implantable cardioverter defibrillator, IVF; idiopathic ventricular fibrillation, OHCA; out of hospital cardiac arrest, SCA; sudden cardiac arrest, UCA: unexplained cardiac arrest; VT; ventricular tachycardia, VF; ventricular fibrillation,.

Table S3. Overview of the diagnostic work-up performed in unexplained cardiac arrest of idiopathic ventricular fibrillation cohorts.

First Author, Year	Performed diagnostic testing												
	Electrical				Imaging					Provocation			
	ECG	Holter	Stress-test	EPS	Echo	CMR	CT	Angiography	CAG	SCB	Isoproterenol	Adrenaline or epinephrine	Ergonovine
Blom, 2019[4]	217 (100%)	121 (56%)	172 (79%)	111 (51%)	208 (96%)	148 (68%)	NA	NA	203 (94%) (or CT)	130 (60%)	NA	NA	53 (24%)
Conte, 2017[5]	100% (by inclusion)	NA	NA	NA	NA	4/8 (50%)	NA	NA	NA	4/8 (50%)	NA	NA	NA
Conte, 2019[6]	100% (by inclusion)	NA	195 (80%)	144 (59%)	100% (by inclusion)	160 (65%)	25 (10%)	NA	220 (90%)	156 (64%)	NA	NA	NA
Chaudhry, 2019[7]	47 (94%)	12 (24%)	30 (60%)	17 (34%)	50 (100%)	27 (54%)	2 (4%)	NA	38 (76%)	9 (18%)	NA	NA	NA
Cunningham, 2020[8]	44 (96%)	NA	19 (41%)	NA	39 (85%)	26 (57%)	1 (2%)	NA	4 (9%)	NA	NA	NA	NA
Frontera, 2019[9]	54 (100%)	NA	45 (83%)	34 (63%)	54 (100%)	38 (70%)	NA	24 (44%)	NA	37 (69%)	38 (70%)	NA	NA
Herman, 2016[1]	100% (by inclusion)	56 (28%)	146 (73%)	62 (31%)	100% (by inclusion)	154 (77%)	NA	8 (4%): RV angiography	100% (by inclusion, with CT/CMR)	131 (66%)	NA	136 (68%)	NA
Jiménez-Jaimez, 2015[10]	100% (by inclusion)	NA	NA	15 (43%)	100% (by inclusion)	13 (37%)	NA	NA	100% (by inclusion)	32 (91%)	NA	35 (100%)	NA

Merghani, 2021[2]	31 (100%)	NA	7 (23%)	NA	31 (100%)	24 (77%)	NA	NA	31 (100%)	25 (81%)	NA	NA	NA
Peres, 2020[11]	NA	NA	NA	NA	NA	NA	NA	NA	NA	NA	NA	NA	NA
Stampe, 2020[12]	81 (100%)	19 (23.5%)	63 (78%)	21 (26%)	81 (100%)	64 (79%)	12 (15%)	NA	72 (89%)	56 (69%)	NA	NA	NA
Stepien-Wojno 2018[13]	44 (100%)	28 (64%)	20 (45%)	NA	44 (100%)	14 (32%)	NA	NA	44 (100%)	5 (11%)	NA	NA	NA
Waldman, 2018[3]	100% (by inclusion)	NA	10 (11%)	NA	100% (by inclusion)	72 (82%)	NA	NA	NA	24 (27%)	NA	13	39
Waldman 2018, IVF only	100% (by inclusion)	6 (12%)	4 (8%)	12 (25%)	100% (by inclusion)	40 (82%)	1 (2%)	5 (10%): RV angiography	47 (95.9%)	21 (43%)	10 (20%)	0 (0)	19 (39%)

Abbreviations: CAG; coronary angiography, CMR; cardiac magnetic resonance CT: computed tomography, ECG; electrocardiogram, EPS; electrophysiological study, NA; not available, SCB; sodium channel blockage, RV; right ventricle.

Table S4. Summary of studies focusing on CMR in patients with ventricular arrhythmias.

First Author, Year, Journal	Setting	Objective	Population	N	Characteristics	CMR protocol	Results	
							**Overall**	**CMR diagnosis**
Andreini, 2020, *JACC Cardiovascular imaging*[14]	Prospective Single center	Ability of CMR to identify a SHD when echocardiography was normal	Patient with a recent diagnosis of a significant VA and no pathological findings on echocardiography	946<br><br>~ 61 sustained VT<br><br>~ 12 cardiac arrest	41 ±16 years, 64% male	1.5T, cine imaging, T1-weighted images, T2-weighted imaging, LGE imaging	- Results not specified between sustained VT or cardiac arrest patients<br>- SHD identified in 241 patients (25.5%)	- 91 myocarditis<br>- 50 ARVC, 5 LDAC<br>- 39 DCM<br>- 22 ischemic heart disease<br>- 12 HCM<br>- 5 non-compaction LV<br>- 5 pericarditis<br>- 10 congenital heart disease
Baritussio, 2017, *Resuscitation*[15]	Retrospective Multicenter (2 centers)	Impact of CMR after clinical, ECG and echocardiogram in patients with an inconclusive angiogram	OHCA patients with inconclusive angiogram (evidence of stable obstructive CAD with no culprit lesion (≥ 70% stenosis) or unobstructed coronaries)	110<br><br>~ 37 stable obstructive CAD<br><br>~ 73 unobstructed coronaries	58 (46-68) years, 76% male	1.5T Cine sequences and post-contrast imaging On indication: T2-short true inversion recovery, and stress perfusion with adenosine	- 72/110 patients with LGE, significantly more in stable obstructive CAD patients<br>- Pathologic substrate identified in 69%<br><br>Echocardiography:<br>- Performed in 92 patients, pathologic substrate in 54%	- 45 IHD<br>- 31 non-IHD<br>- 9 nonspecific findings<br>- 25 structurally normal heart<br><br>- Same diagnosis as echocardiography in 51/92 patients<br>- CMR changed diagnosis in 27 patients (25%); more in stable obstructive CAD patients (based on LGE)
Hennig, 2018, *Europace*[16]	Prospective, single center	Assess the diagnostic yield of CMR (including high-resolution LGE) after non-CMR diagnostic workup	Patients with first episode of (sustained or non-sustained) VT, VF or aborted SCD	157<br><br>~ 52 NSVT, 88 sustained VT<br><br>~17 VF or aborted SCD	54±17 years, 75% male	1.5T, cine imaging, LGE imaging including high resolution LGE imaging	- CMR identified a structural substrate in 105 patients; in 12/17 (71%) patients with VF/SCD	- 46 ICM<br>- 38 NICM<br>- 11 ARVC<br>- 3 HCM<br>- 7 Other<br>- 52 no SHD

				~45 patients with a known SHD before inclusion				- Pre-CMR diagnostic work-up identified a SHD in 28/112 patients, increasing this rate to 60/112 after including CMR results<br>- CMR modified the diagnostic category in 48/112 patients
Kim, 2021, *JCM*[17]	Retrospective Multicenter (3 centers)	Evaluate the usefulness of CMR imaging in OHCA survivors with inconclusive CAG findings and treatment with TTM	Unexplained OHCA with targeted temperature and inconclusive CAG, excluded patients with proven etiology by CAG (culprit lesion), ergonovine stress test, EPS or echocardiography, diagnosed (and excluded) patients with IVF before CMR.	40	Age 37.5 years (IQRS 29.0-51.3), 72.5% men	1.5T, cine images, T1-weighted imaging, T2-weighted imaging, LGE	- 23 (57.5%) normal<br>- 12 (30%) non-diagnostic findings (wall motion dyskinesia, decreased contractility)	- 23 normal<br>- 12 non-diagnostic<br>- 5 non-ICM
Neilan, 2015, *JACC Cardiovascular Imaging*[18]	Prospective (collected data) and retrospective (follow up adverse events)<br><br>Multicenter (2 centers)	Identify the role of LGE CMR in the work-up of patients with SCA	SCA (either VT or VF) survivors referred for CMR after clinical assessment, ECG, echocardiogram and coronary angiogram without clear cause	137<br><br>~105 VF	Age 56±12 years, 66% male	1.5T or 3T, ECG gating, breath-holding, cine steady-state free precession imaging and LGE imaging.	- LGE present in 98 (71%)<br>- VT was more common in patients with LGE, VF in patients without LGE<br>- Diagnosis or potential substrate identified in 104 patients (76%)	- 60 infarct pattern LGE<br>- 21 non-infarct pattern LGE<br>- 14 active myocarditis<br>- 3 HCM<br>- 3 Sarcoidosis<br>- 3 ARVC

							- The presence and extent of LGE were associated with adverse events	
Marstrand, 2016, *Europace*[19]	Retrospective<br><br>Single center	Assess the diagnostic precision of CMR and the ability to change the indication for family screening	Cardiac arrest, VT or syncope patients referred for CMR	79<br><br>~ 31 VT<br><br>~ 44 aborted SCD	43 (13) years, 71% male = aborted SCD/VT population	1.5 or 3T, steady-state free precision cine images, LGE by T1-weighted gradient echo images, T2-weighted images at physician's discretion	- Based on CMR data, preliminary diagnoses were changed in 38 of 79 patients, decreasing the indication for family screening with 19%	- 29 idiopathic VF/VT<br>- 9 ARVC<br>- 3 DCM<br>- 2 HCM<br>- 6 unspecified cardiomyopathy<br>- 9 IHD<br>- 5 other<br>- 4 primary electrical cardiac diseases<br>- 3 sarcoidosis/myocarditis<br>- 9 normal (only in NSVT/syncope group)
Rodrigues, 2017, *Circulation: Cardiovascular Imaging*[20]	Retrospective<br><br>Multicenter	Determining the pathogenesis and prognosis with CMR	Cardiac arrest survivors or hemodynamically unstable sustained VT, without CAD (on CAG, stenosis ≥30% excluded) who underwent CMR.	164<br><br>~ 135 SCA	48±15yrs, 65% men	1.5T, cine images, LGE images, and LGE. On indication: T1-weighted and T2-weighted	- Contributed to the diagnosis in 80 patients (49%)<br>- 50 patients in whom the final diagnosis would not been made without CMR (30%)<br>- Abnormal LGE present in 61 (37%)<br>- CMR-defined diagnosis, presence and extent of LGE were associated with more MACE (41 vs 21)	- 27 DCM<br>- 22 myocarditis/sarcoidosis<br>- 13 occult MI<br>- 9 HCM<br>- 3 ARVC<br>- 4 severe valvular disease<br>- 1 Takotsubo<br>- 1 undetermined CMP/other<br>- 30 minor/nonspecific<br>- 55 normal

White, 2012, *Cardiovasc Imaging*[21]	Prospective<br><br>Single center	Diagnostic yield of CMR imaging compared to non-CMR imaging	Patients with aborted SCD or symptomatic SMVT, excluded patients with ischemic events	82<br><br>~30 aborted SCD patients<br>~22 VF	46 ±14 years, 70% male (SCD patients)	3T (n=52), 1.5T (n=28), cine imaging (steady state free precession-base pulse sequence), T2-weighted imaging, DE imaging, T1-weighted imaging	- CMR identified substrate for VA in 61 patients (74%); 20 of the 30 patients with SCD<br>- New or alternative diagnosis in 41 (49%) of the patients<br>- Change in diagnosis in SCD: 21 (68%)	- 21 normal<br>- 5 unexplained LV dysfunction<br>- 29 CAD with LV dysfunction<br>- 17 inflammatory CMP<br>o 3 sarcoidosis<br>- 1 HCM<br>- 9 Other<br>o 6 ARVC<br>o 1 LVNC<br>o 2 unknown midwall fibrosis
Zaremba, 2018, *European Journal of Radiology*[22]	Retrospectiv e<br><br>Single center	CMR findings in young aborted SCD patients without ischemic cause	First episode of aborted SCD or sustained VT, patients <50 years, who underwent CMR and had ICD implantation<br><br>Excludes reversible causes with ECG, blood test, echocardiograph y and CAG.<br><br>Patients with CHD, myocarditis, previous structural heart disease, electrolyte abnormality or drug intoxication were excluded	36<br><br>~ 20 VF as initial rhythm	37.6 years (24.1-43.2), 69% male	1.5T (n = 24), 3T (n = 10), 1.5T (n=2), cine images, LGE imaging	- CMR did not alter the diagnosis in any study participants<br>- LGE was detectable in 87%, but only in small amounts and did not differ between CMP and arrhythmia patients<br>- Aborted SCD survivors had impaired global strain (longitudinal, circumferential and radial direction) compared to reference data, but was not associated with cardiomyopath y	- Diagnosis was not solely made on CMR

Zorzi, 2018, *Heart Rhythm* [23]	Retrospective Single center	Determine the diagnostic and prognostic role of CMR in OHCA<br><br>Compares patients with CAD and without	Survivors of arrhythmic OHCA who had CAG and CMR ≤ 7 days	44	Age 43 (39-62) years, 84% male	Cine imaging, T2-weighted imaging, LGE imaging	- 41% had CAD (n = 18)<br>- Pathologic myocardial substrate in 19 (73%) patients without CAD<br>- CMR confirmed diagnosis in all CAD patients and modified the initial clinical diagnosis in 11 patients (42%) and confirmed in 58% of patients without CAD<br>- Myocardial edema suggest a potentially reversible myocardial lesion with no arrhythmic event in 3 years of follow up	- 5 DCM<br>- 3 MVP<br>- 2 IHD<br>- 2 non-ischemic LV scar<br>- 1 ACM<br>- 1 HCM<br>- 1 Takotsubo<br>- 4 acute myocarditis<br>- 7 structural normal heart
Zorzi, 2021, *JAHA* [24]	Retrospective Multicenter (9 centers)	Investigate the possible favorable prognosis of myocardial oedema in SCA patients	SCA survivors who underwent CMR imaging < 1 month with T2-wighted imaging and ICD implantation	101	74 (31-59) years, 70% male	1.5T, cine imaging, T2-weighted imaging, LGE imaging	- Myocardial edema present in 18/101 patients; 7 non-ischemic and 11 ischemic<br>- CMR identified a myocardial substrate in 43 SCA survivors (non-ischemic)	- 29 normal structural heart<br>- 13 DCM<br>- 4 ACM<br>- 3 HCM<br>- 16 isolated non-ischemic scar<br>- 7 MVP<br>- 7 acute myocarditis<br>- 21 IHD

Abbreviations: ACM: arrhythmogenic cardiomyopathy, ARVC; arrhyhtmogenic right ventricular cardiomyopathy, CAG; coronary angiogram, CHD: chronic heart disease, CMP; cardiomyopathy, CMR; cardiac magnetic resonance, DCM; dilated cardiomyopathy, ECG: electrocardiogram, EPS: electrophysiological study, HCM; hypertrophic cardiomyopathy, FU; follow up, IHD: ischemic heart disease, ICM; ischemic cardiomyopathy, LDAC; left dominant arrhythmogenic cardiomyopathy, LGE; late gadolinium enhancement, LV; left ventricle, LVEF: left ventricular ejection fraction, LVNC: left ventricular noncompaction, MACE: major adverse cardiac events, MI; myocardial infarction, MVP; mitral valve prolapse, NICM; non-ischemic cardiomyopathy, NSVT: non-sustained ventricular tachycardia, OHCA: out of hospital cardiac arrest, SCAD;

stable obstructive coronary artery disease, SCD; sudden cardiac death, SHD; structural heart disease, SMVT; sustained monomorphic VT, TTM; targeted temperature management, VA; ventricular arrhythmias, VF; ventricular fibrillation, VT; ventricular tachycardia.

List of included studies

1. Herman, A. R. M. *et al.* Outcome of apparently unexplained cardiac arrest: Results from investigation and follow-up of the prospective cardiac arrest survivors with preserved ejection fraction registry. *Circ. Arrhythmia Electrophysiol.* **9**, 1–10 (2016).
2. Merghani, A. *et al.* Diagnostic impact of repeated expert review & long-term follow-up in determining etiology of idiopathic cardiac arrest. *J. Am. Heart Assoc.* **10**, (2021).
3. Waldmann, V. *et al.* Characteristics and clinical assessment of unexplained sudden cardiac arrest in the real-world setting: Focus on idiopathic ventricular fibrillation. *Eur. Heart J.* **39**, 1981–1987 (2018).
4. Blom, L. J. *et al.* Incidence and predictors of implantable cardioverter-defibrillator therapy and its complications in idiopathic ventricular fibrillation patients. *Europace* **21**, 1519–1526 (2019).
5. Conte, G. *et al.* True idiopathic ventricular fibrillation in out-of-hospital cardiac arrest survivors in the Swiss Canton Ticino: Prevalence, clinical features, and long-term follow-up. *Europace* **19**, 259–266 (2017).
6. Conte, G. *et al.* Out-of-hospital cardiac arrest due to idiopathic ventricular fibrillation in patients with normal electrocardiograms: Results from a multicentre long-term registry. *Europace* **21**, 1670–1677 (2019).
7. Chaudhry, U. *et al.* Idiopathic ventricular fibrillation – Long term prognosis in relation to clinical findings and ECG patterns in a Swedish cohort. *J. Electrocardiol.* **56**, 46–51 (2019).
8. Cunningham, T. *et al.* Initially unexplained cardiac arrest in children and adolescents: A national experience from the Canadian Pediatric Heart Rhythm Network. *Hear. Rhythm* **17**, 975–981 (2020).
9. Frontera, A. *et al.* Long-Term Follow-Up of Idiopathic Ventricular Fibrillation in a Pediatric Population: Clinical Characteristics, Management, and Complications. *J. Am. Heart Assoc.* **8**, (2019).
10. Jiménez-Jáimez, J. *et al.* Diagnostic Approach to Unexplained Cardiac Arrest (from the FIVI-Gen Study). *Am. J. Cardiol.* **116**, 894–899 (2015).
11. Peres, N. *et al.* Comprehensive assessment of unexplained sudden cardiac death and idiopathic ventricular fibrillation during the index hospitalization: A systematic thorough investigation to unmask specific etiology. *Arch. Cardiovasc. Dis. Suppl.* **12**, 106–107 (2020).
12. Stampe, N. K. *et al.* Clinical characteristics and risk factors of arrhythmia during follow-up of patients with idiopathic ventricular fibrillation. *J. Cardiovasc. Electrophysiol.* **31**, 2677–2686 (2020).
13. Stępień-Wojno, M. *et al.* Sudden cardiac arrest in patients without overt heart disease: A limited value of next generation sequencing. *Polish Arch. Intern. Med.* **128**, 721–730 (2018).
14. Andreini, D. *et al.* CMR for Identifying the Substrate of Ventricular Arrhythmia in Patients With Normal Echocardiography. *JACC Cardiovasc. Imaging* **13**, 410–421 (2020).
15. Baritussio, A. *et al.* Out of hospital cardiac arrest survivors with inconclusive coronary angiogram: Impact of cardiovascular magnetic resonance on clinical management and decision-making. *Resuscitation* **116**, 91–97 (2017).
16. Hennig, A. *et al.* High-resolution three-dimensional late gadolinium-enhanced cardiac magnetic resonance imaging to identify the

underlying substrate of ventricular arrhythmia. *Europace* **20**, f179–f191 (2018).
17. Kim, S. M. *et al.* Cardiac magnetic resonance imaging for nonischemic cardiac disease in out-of-hospital cardiac arrest survivors treated with targeted temperature management: A multicenter retrospective analysis. *J. Clin. Med.* **10**, 1–11 (2021).
18. Neilan, T. G. *et al.* Late gadolinium enhancement among survivors of sudden cardiac arrest. *JACC Cardiovasc. Imaging* **8**, 414–423 (2015).
19. Marstrand, P. *et al.* Cardiac magnetic resonance imaging After ventricular tachyarrhythmias increases diagnostic precision and reduces the need for family screening for inherited cardiac disease. *Europace* **18**, 1860–1865 (2016).
20. Rodrigues, P. *et al.* Diagnosis and Prognosis in Sudden Cardiac Arrest Survivors Without Coronary Artery Disease: Utility of a Clinical Approach Using Cardiac Magnetic Resonance Imaging. *Circ. Cardiovasc. Imaging* **10**, 1–9 (2017).
21. White, J. A. *et al.* Utility of cardiovascular magnetic resonance in identifying substrate for malignant ventricular arrhythmias. *Circ. Cardiovasc. Imaging* **5**, 12–20 (2012).
22. Zaremba, T., Brøndberg, A. K., Jensen, H. K. & Kim, W. Y. Cardiac magnetic resonance characteristics in young survivors of aborted sudden cardiac death. *Eur. J. Radiol.* **105**, 141–147 (2018).
23. Zorzi, A. *et al.* Diagnostic value and prognostic implications of early cardiac magnetic resonance in survivors of out-of-hospital cardiac arrest. *Hear. Rhythm* **15**, 1031–1041 (2018).
24. Zorzi, A. *et al.* Prognostic role of myocardial edema as evidenced by early cardiac magnetic resonance in survivors of out-of-hospital cardiac arrest: A multicenter study. *J. Am. Heart Assoc.* **10**, 1–13 (2021).
